# *PLOS Digital Health*, a new journal driving transformation in the delivery of equitable and unbiased healthcare

**DOI:** 10.1371/journal.pdig.0000009

**Published:** 2022-01-18

**Authors:** Leo Anthony Celi

**Affiliations:** PLOS Digital Health, San Francisco, California, United States of America

The promise of artificial intelligence (AI) has propelled digital health to the forefront of the medical field. The zettabytes of data captured by digital health tools and systems present an opportunity to retool the medical knowledge system such that it represents the majority of the world’s population who are typically excluded from clinical trials and observational studies.

But the race to provide data upon which innovations are designed has left many groups behind, particularly those from low- and middle-income countries (LMICs). LMICs struggle to publish research and generate data at the same rate as their high-income counterparts. This is primarily because of inadequate research funding and support and a limited capacity to digitize and analyze data.

If this race is left to run its own course, innovations will be built on data that disproportionately reflects a majoritized population. Likewise, it stands to reason that only members of this group will stand to benefit. Instead of addressing and preventing existing and well-documented disparities in healthcare, these disparities will simply be encrypted and perpetuated. Differential accuracy based on race has already been noted in some machine learning–based healthcare algorithms, potentially affecting the quality of care that nonwhite populations receive [1]. Therefore, the role of peer-reviewed journals in this campaign cannot be understated. At *PLOS Digital Health*, we will uphold the following principles.

We promote the diversification of author lists and spectrum of research questions to better represent the perspectives and concerns of minority populations and LMICs. Parachute research must be eliminated, and some journals have already taken concrete steps to achieve this. *The Lancet Global Health* declared it “looks extremely unfavorably on papers submitted by authors who have done primary research in another country (particularly a low-income or middle-income country) but not included any author from that nation” [2], and journals from publishers including Nature, Cell, and Wiley have supported and instituted reflexivity statements [3,4,5]. *PLOS* has also integrated a mandatory questionnaire for submissions presenting research done in other countries. This questionnaire investigates the involvement and participation of local community members throughout the research process and explores how the results and possible benefits of the research will be shared.

Capacity building and participatory research among minoritized groups are crucial. The Fogarty International Center of the US National Institutes of Health [6] and the UK National Institute for Health Research [7] enact funding programs that categorically focus on strengthening the research capacity of LMICs and the training of local researchers. This support will increase LMIC representation in the scientific community through journal publications. More importantly, capacity building and training will create local centers of scientific excellence and build sustainable research environments in LMICs [6]. The Bridge2AI and the AIM-AHEAD initiatives of the NIH seek to generate datasets and diversify the research community in order to ensure that the benefits of digital health are indeed equitable.

Historically, the centralized opacity of journals creates bias toward the types of papers and authors accepted [8]. At *PLOS Digital Health*, we are invested in transforming the governance within peer-reviewed journals to better serve the translation of medical knowledge into public goods.

Scientific integrity and progress are at the forefront of this journal. However, we believe that aggressive changes to the scientific process are imperative if we aim to address the decades-old problems that negatively shape the direction of academic knowledge [9].

The problems that persist in medicine today—the unequal map of the knowledge system, disparities in health outcomes and health data, and a propensity to exclude—are partly a result of decisions made by academic journal publications. However, in the realm of digital health, it remains to be seen whether these issues will be amplified or subdued. Academia must strengthen diversity and inclusivity among its leaders as well as among those who contribute their thoughts and perspectives in the form of publication. Likewise, we aim to foster a culture of equity, innovation, and excellence, and as we move forward.
